# Supplementary material for: Spindle assembly checkpoint-dependent mitotic delay is required for cell division in absence of centrosomes
Source: eLife. 2024 Aug 2;12:RP84875. doi: 10.7554/eLife.84875 (PMC11296703; doi:10.7554/eLife.84875)
Supplement: Figure 1—figure supplement 3—source data 1. [file elife-84875-fig1-figsupp3-data1.zip › Figure1-supplement3-source data 1/Blot_SupplementalFigure3c.pdf]

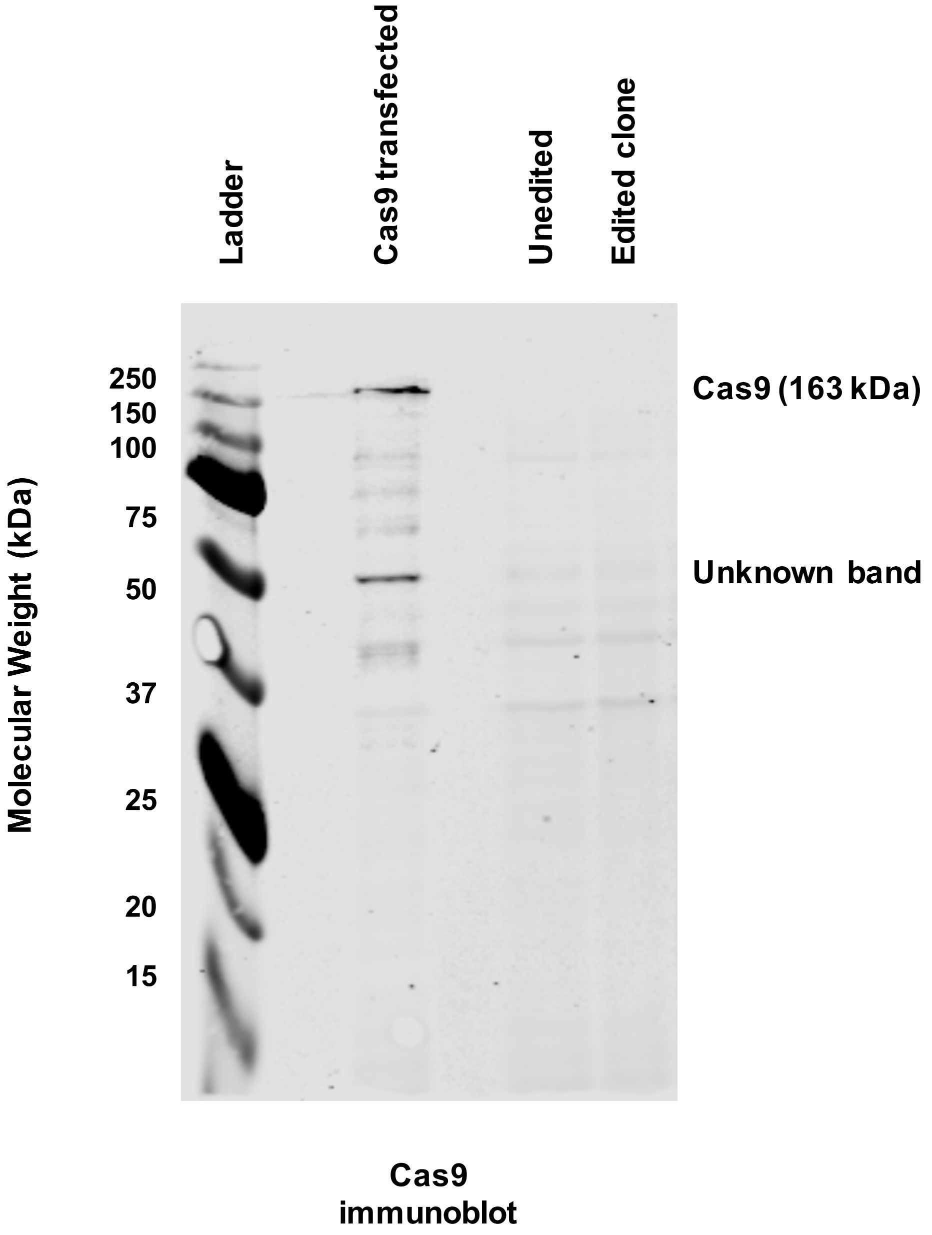

Ladder
Cas9 transfected
Unedited
Edited clone
Molecular Weight (kDa)
250
150
100
75
50
37
25
20
15
Cas9 (163 kDa)
Unknown band
Cas9
immunoblot
