# Supplementary figures and images for: Spindle assembly checkpoint-dependent mitotic delay is required for cell division in absence of centrosomes
Source: eLife. 2024 Aug 2;12:RP84875. doi: 10.7554/eLife.84875 (PMC11296703; doi:10.7554/eLife.84875)

Size (kilobasepairs)

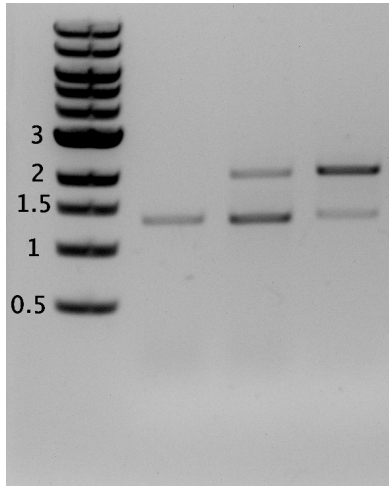

*mEGFP-TUBA1B*

Unedited *TUBA1B*

Supplement: Figure 1—figure supplement 3—source data 1. [file elife-84875-fig1-figsupp3-data1.zip › Figure1-supplement3-source data 1/Figure1 supplement3a_DNAgel_labelled.pdf]

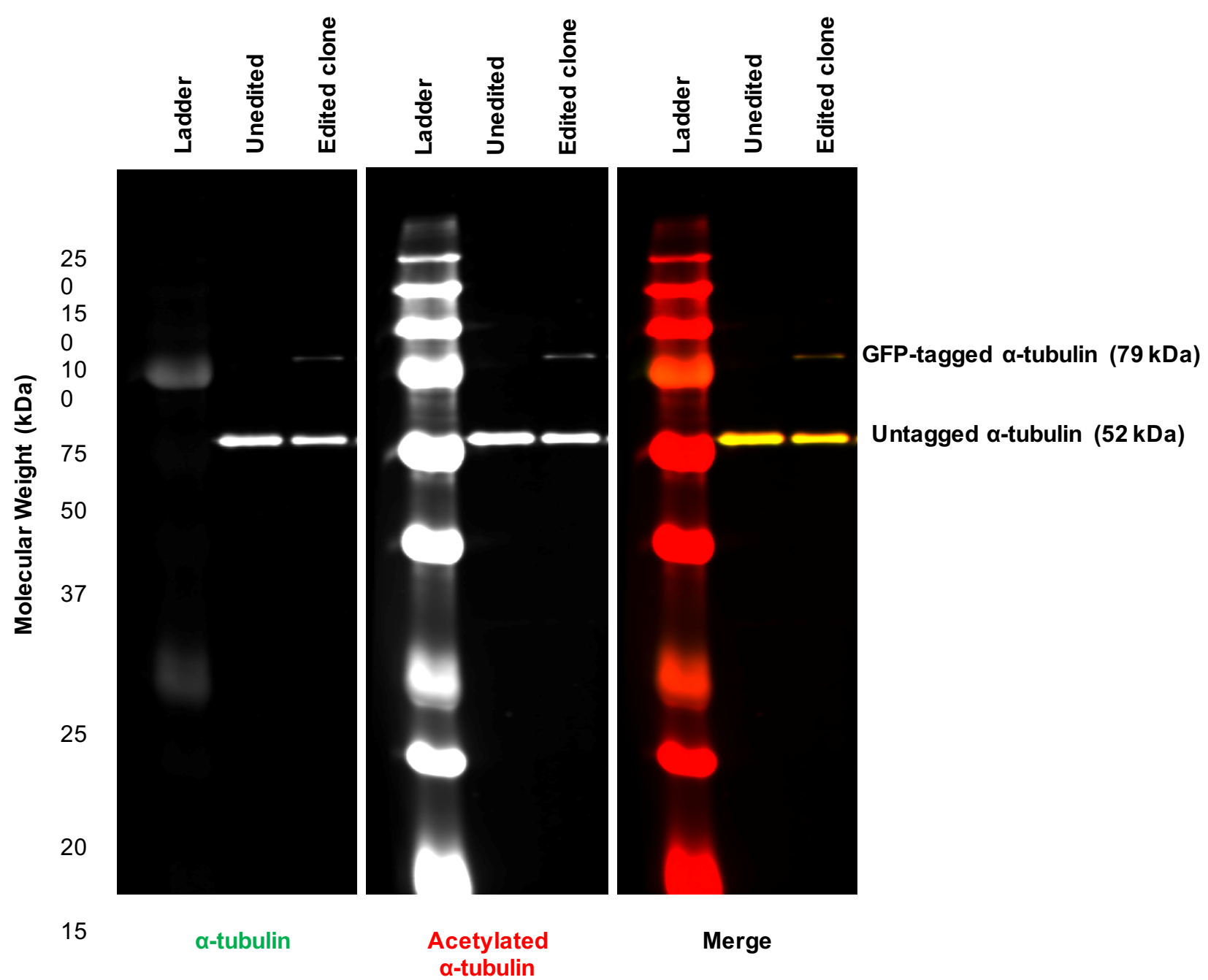

Supplement: Figure 1—figure supplement 3—source data 1. [file elife-84875-fig1-figsupp3-data1.zip › Figure1-supplement3-source data 1/Blot_SupplementalFigure3d.pdf]

Molecular Weight (kDa)

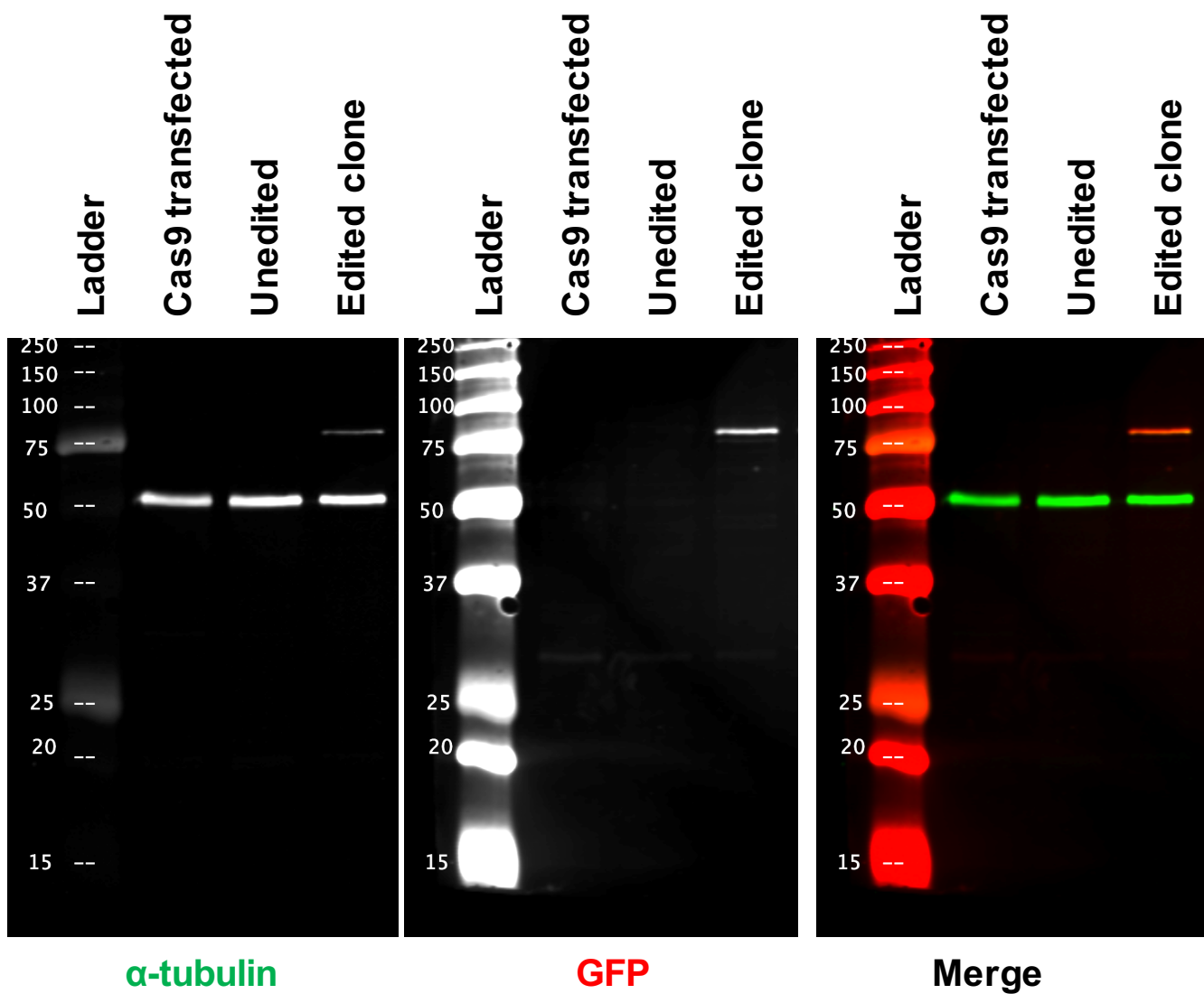

GFP-tagged  $\alpha$ -tubulin (79 kDa)

Untagged  $\alpha$ -tubulin (52 kDa)

Supplement: Figure 1—figure supplement 3—source data 1. [file elife-84875-fig1-figsupp3-data1.zip › Figure1-supplement3-source data 1/Blot_SupplementalFigure3b.pdf]

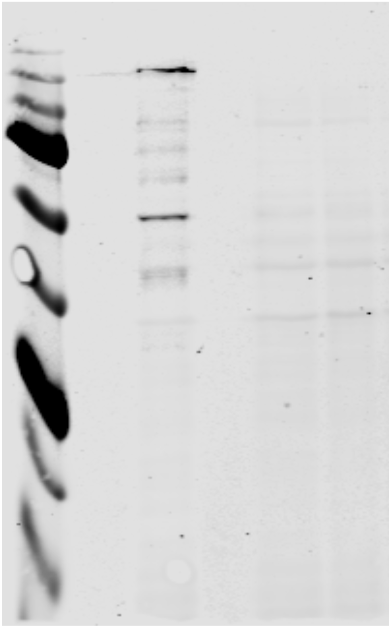

Supplement: Figure 1—figure supplement 3—source data 2. [file elife-84875-fig1-figsupp3-data2.zip › Figure1-supplement3-source data 2/Figure1 supplement3c_Cas9_blot_raw.png]

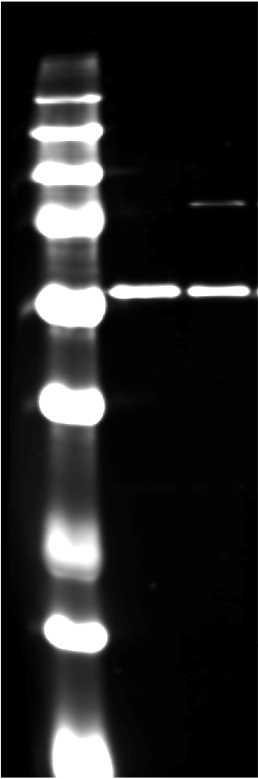

Supplement: Figure 1—figure supplement 3—source data 2. [file elife-84875-fig1-figsupp3-data2.zip › Figure1-supplement3-source data 2/Figure1 supplement3d_AcTub_blot_raw.png]

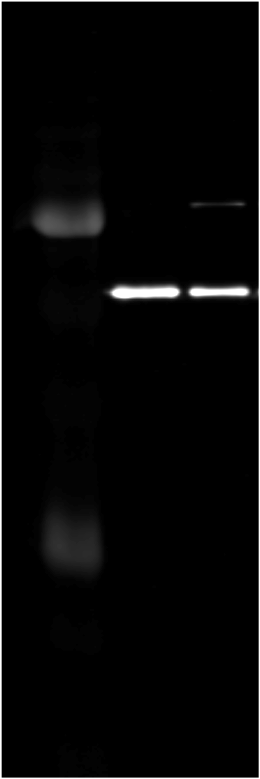

Supplement: Figure 1—figure supplement 3—source data 2. [file elife-84875-fig1-figsupp3-data2.zip › Figure1-supplement3-source data 2/Figure1 supplement3d_aTub_blot_raw.png]

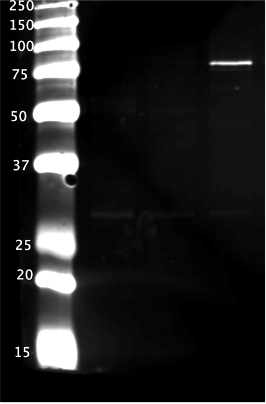

Supplement: Figure 1—figure supplement 3—source data 2. [file elife-84875-fig1-figsupp3-data2.zip › Figure1-supplement3-source data 2/Figure1 supplement3b_GFP_blot_raw.png]

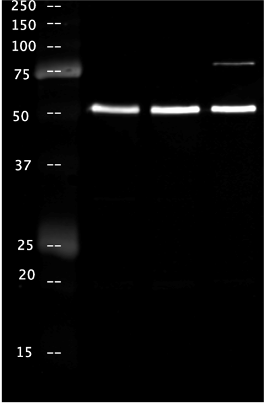

Supplement: Figure 1—figure supplement 3—source data 2. [file elife-84875-fig1-figsupp3-data2.zip › Figure1-supplement3-source data 2/Figure1 supplement3b_aTub_blot_raw.png]

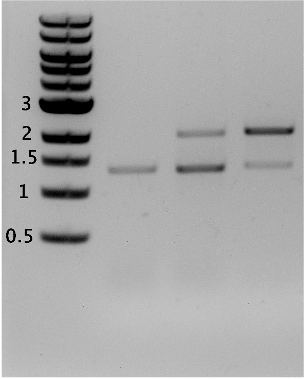

Supplement: Figure 1—figure supplement 3—source data 2. [file elife-84875-fig1-figsupp3-data2.zip › Figure1-supplement3-source data 2/Figure1 supplement3a_DNAgel_raw.png]

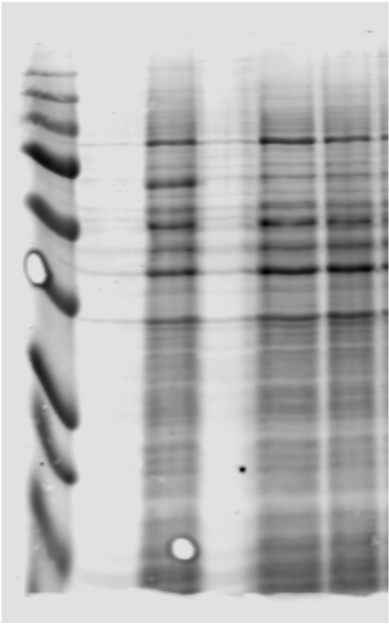

Supplement: Figure 1—figure supplement 3—source data 2. [file elife-84875-fig1-figsupp3-data2.zip › Figure1-supplement3-source data 2/Figure1 supplement3c_REVERT_blot_raw.png]
